# Age Differences in Work Stress, Exhaustion, Well-Being, and Related Factors From an Ecological Perspective

**DOI:** 10.3390/ijerph16010050

**Published:** 2018-12-25

**Authors:** Hui-Chuan Hsu

**Affiliations:** School of Public Health, Taipei Medical University, Taipei 11031, Taiwan; gingerhsu@seed.net.tw; Tel.: +886-2-2736-1661-6524

**Keywords:** age difference, exhaustion, well-being, work stress, work environment

## Abstract

The aim of this study was to examine the association of work stress, exhaustion, well-being, and related individual, organizational, and social factors, focusing especially on age differences in Taiwan. The data were from the 2015 Taiwan Social Change Survey. The participants were community-based adults, aged 18 years or older, selected via stratified multistage proportional probability sampling from the Taiwanese population. Well-being was measured by self-rated health and psychological health. Descriptive analysis, one-way analysis of variance, and linear regression analysis were used. Work stresses were related to three types of exhaustion, and exhaustion was related to well-being. Individual working style (being creative and using new methods), organizational factors (job satisfaction, work-family conflict, discrimination against women), and social factors (difficult finding a good job than older cohorts) were related to well-being. Older age was related to worse self-rated health, and age showed a reverse-U-shaped relation with psychological health. The resilience of older workers could be an opportunity for the global active aging trend, and interventions to support older workers in organizations would be beneficial.

## 1. Introduction

Work stress and its impact on exhaustion and well-being have been an emerging issue in health-related research. Long working hours, or overwork, and high job strain or occupational burnout have been found to be related to cerebrovascular disease [1], the incidence of diabetes, and even uncontrolled eating disorders [2,3]. Past research has typically focused on organizational- and individual-level factors, such as the demand-control model [4], or the effort-reward imbalance model [5]. Job strain and stress are found to be related to emotional exhaustion and depersonalization [6,7], and could affect anxiety and depression [8] or other psychiatric morbidities [9,10]. However, rapid aging has had an enormous influence on the labor environment, organizations, and society.

As population aging becomes a global trend, exhaustion and prolonged working age have been issues for older workers [11,12]. Active aging is expected not only to prolong working years but also to increase age integration and reduce intergenerational conflicts [13,14]. The willingness to work and the effects of working ability on health and well-being could be the core issues for an aging society. However, whether the negative impacts of exhaustion on well-being can be reduced or avoided via individuals’ working methods or attitudes, or by systemic and policy changes for both younger and older workers has been little explored.

### 1.1. Theoretical Explanations

Theoretical models have been developed to explain the relation between work stress and exhaustion. The demand-control theory [4] suggests that a sense of control over one’s job can buffer the impact of job demands and increase job satisfaction. When effort and reward are imbalanced at work, adverse health effects arise. The key to job stress is to increase workers’ sense of control, including providing resources, promoting self-efficacy and active coping methods, and social support. The effort-reward imbalance model [5] is based on the reciprocity of exchange. Psychosocial factors, such as emotional demands, the demands of hiding emotions, sensorial demands, the meaning of work, commitment to the workplace, organizational influence, trust, the social community at work, leadership quality, predictability, role clarity, work-life balance, and negative acts (e.g., violence, and bullying), are important [15].

Cortisol reactivity is related to reactions to stress. When there is a moderate level of stress, the regulation mechanism is at its best, which explains the enhanced resilience. However, cortisol reactivity [16] is like a U-shaped reaction. Too much stress causes neuro-endocrine effects. Stressors come from life events or chronic stressors [17], and work stress is usually considered a chronic stressor. In the long run, physical health and psychological health are affected, with additional impacts on psychological well-being, on performance and willingness to work.

According to Bronfenbrenner’s ecological system perspective [18], humans are affected by the values and beliefs from their microsystem (individual factors), mesosytem (interpersonal factors), exosystem (organizational factors), macrosystem (policy or system factors), and even ecological transitions (cohort effects and life course). This ecological perspective can be applied to explain the factors related to work and well-being for workers due to individual, interpersonal, organizational, and social factors. The cohort differences could explain the ecological transitions in work and well-being. The purpose of this study was to examine the effects of different factors from an ecological perspective on work stress, exhaustion, and well-being, especially in relation to age differences.

### 1.2. Work Stress, Exhaustion, and Organizational Factors

Working organizational factors, including labor policies, working conditions, interpersonal support, and even workplace leadership and management, affect the workload, stress, and exhaustion levels of workers. Lower levels of job control and decreased social support at work are related to a higher risk of dementia [19]. Lower job demands and physical workload, high task resources, and good leadership are related to better work ability. Pisanti et al. [20] supports the Job Demand Control Support model, and the occupational coping self-efficacy buffers the stress. Further, by a longitudinal study on nurses, burnout and social support predict emotional exhaustion and depersonalization, while burnout, demand, and control predict personal accomplishment [8]. Working engagement, better lifestyle (such as exercise, good sleep, non-smoking), low demanding job, low physical workload, and high task resources are related to better working ability [21]. The study by Turnell et al. [22] also supports the job demand-resources model: Lower job resource and higher job demands are related to greater burnout. Higher job resource is related to higher engagement.

Higher levels of social support from supervisors and coworkers, greater control over one’s job, feedback, and autonomy are moderately strongly related to lower levels of emotional exhaustion. A meta-analysis study showed that higher job support and higher job control are moderately strongly related to lower emotional exhaustion [23]. Job demands are negatively related to psychological health, but the coping resources buffer the job demands on psychological health and further on turnover [24]. Emotional exhaustion is related to lifestyle, role overload/role ambiguity/role boundary. Job strain, personal strain, and personal resources are related to emotional exhaustion, but only job and personal strains are related to burnout [6]. Crawford et al. [25] found that resources may reduce burnout. Furthermore, challenging job demands are positively related to engagement as well as burnout, while hindrance demands are positively related to burnout but negatively related to engagement. The personal and social capital in work would affect the job stress perception, and then affect life satisfaction [26]. That implies we may not change personality, but we can change the working environment and work capital to reduce occupational stress.

### 1.3. Work Stress, Exhaustion, and Individual Factor

Personality, lifestyle, working style, work capital, and demographic characteristics can be related to the perception of stress and exhaustion. Better lifestyle is related to better working ability [21]. In addition, work–family conflict and role ambiguity or conflict have been connected with work stress and emotional exhaustion [6,27]. Broader sources of social support are related more strongly to work-family conflicts [28]. Social support from family is also important. Lee et al. [29] found that the social support from family and from the supervisor may buffer the emotional exhaustion. However, work-family conflict (both work interference with family, and family interference with work) are related to emotional exhaustion.

Factors that can moderate the influence of work stress on exhaustion and well-being include demographics, resilience, personality, self-efficacy, and coping styles. Higher burnout is found to be related to younger age, race and occupation, financial strain, and health status [30]. When people have higher resilience, their psychological health is better, even in high-stress occupations, and they experience lower rates of burnout [7]. Athlete’s resilience and coach’s social support moderated the stress-burnout effect [31]. Resilience is related to psychological health for a high-stress occupation such as firefighters, and the social support from bosses and the emotional demand show that interaction affects resilience [32]. Personality is related to exhaustion, too [33]. Having greater self-efficacy and using positive coping strategies can help to reduce stress and burnout, but not all coping strategies work [34,35,36,37].

### 1.4. Aging and Work

Under the tsunami of global aging, active aging has been promoted [38], and thus aging effects for older workers are necessary to explore. Older workers can face more barriers and stressors at work, such as physical strength limitations and health concerns, gaps related to using new technology, and the engagement in work. Health has been proven to be related to retirement or exit from labor force, including physical health and mental health [39,40,41]. Guglielmi et al. [42] examined the gain cycle from work demands to job satisfaction, and younger workers respond better than older workers. For those older (aged 65 or more) workers whose work was in low control, less effort–reward imbalanced work and having poorer health were less likely to work in the old age. This suggests that the work ability and work condition determine the participation in work for older adults. Older workers also have their own expectation of retirement age, and the closer to their planned retirement age, the more likely they were to disengage from work [43].

However, the barriers about aging depend on job characteristics, and aging is not necessarily to be a barrier. Blue-collar workers were less likely still working at age 65, while white-collar workers were more likely to continue work [44]. Older workers are more resilient than younger ones against work-family conflicts for academic employees, and older workers are more capable at buffering workload stress and life satisfaction in service sectors [45]. Older workers seem to have higher emotional suppression at work [46,47]. Jason et al. [48] used the socio-ecological model to examine the multiple chronic conditions and resilience effects on workforce transition in late life. By using the longitudinal two-wave data, resilience buffered the negative effects of multiple chronic conditions on workforce engagement and remained independent. That implies that having higher resilience would help maintain work engagement for older workers. 

The perception towards older workers or age discrimination may affect the willingness of work behavior or the psychological state in working [49]. Although ageism may exist in the workplace, a meta-analysis study found that perceptions regarding older workers are varied, i.e., not entirely positive or negative [50].

For older workers, environmental factors, physical factors, work rhythm, working relationships, and work characteristics are all related to the perception of one’s ability to continue to work [51]. Some work strategies help older workers age successfully. Security, relationship development, continuous learning, and career management strategies predict perceived success at work [52].

### 1.5. Background on Taiwan

Taiwan has been an aging society since 1994. Unlike the response of rapidly growing industries and a well-developed health policy to face the aging trend, labor policy has remained relatively conservative. The mandated retirement age is 65 years old for the public sector, although people can work in the private sector until they are 70 years old. In 2014, the labor participation rates for individuals aged 55–59, 60–64, and 65 years and older were 54.4%, 35.6%, and 8.7%, respectively [53]. The average working time per year in 2016 was 2034 hours, which is much higher than in the Organization for Economic Co-operation and Development countries [54]. Older cohorts of workers must face the challenge of new work-related skills and technology, and younger cohorts of workers seem trapped in a low-salary working environment. The incidence of work-related exhaustion has increased in recent years. Improving employment and delaying retirement to encourage active aging for older workers are emerging issues in Taiwan.

## 2. Materials and Methods

### 2.1. Data and the Sample

Data were obtained from the Work Orientation module of the 2015 Taiwan Social Change Survey. The respondents included community-based adults 18 years or older, selected via stratified multistage probability proportional sampling from the Taiwanese population. These secondary data were anonymous when provided by the Survey Research Data Archive of Academia Sinica [55]. The original sample size was 2031, but only those who were working were included for analysis in this study; a total of 1298 participants. The study received approval from the Medical Research Ethics Committee beforehand.

### 2.2. Measures

#### 2.2.1. Subjective Well-Being 

Subjective well-being was measured via five items connected to self-rated health and psychological health. Self-rated health asked participants to rate their health (both physically and psychologically) from poor to excellent (scored 1–5). The three psychological health items asked respondents to indicate the frequency, in the past month, of their experience of specific feelings: Calm, energetic, or depressive/down (reverse scoring). The score ranged from 1 to 5, or never to always, respectively. The Cronbach’s alpha of the 3 items of psychological health was 0.699.

#### 2.2.2. Work Exhaustion

Work exhaustion was measured by three items: How often do you feel physically exhausted? How often do you feel emotionally exhausted? How often do you feel you cannot stand it? The score was from 1 to 5, or never (1) to always (5). The Cronbach’s alpha of the 3 items of work exhaustion was 0.827.

#### 2.2.3. Work Stress

Work stress was measured by six items indicating the frequency of the following work situations: The work is physically demanding, the work is stressful, it is possible to work at home on weekdays, usually needing to work on weekends, feeling tired when thinking of work, and thinking of work while going to sleep. The score was from 1 to 5, never (1) to always (5). The Cronbach’s alpha of the six kinds of work stress = 0.486, indicating moderate associations of the items. Total work stress was the sum of six items, scored from 6 to 30.

#### 2.2.4. Demographics

The demographic variables included age (18–39, 40–54, 55–64, 65–74, and 75+), gender (male = 1, and female = 0), education (ordinal score from 1 to 7, indicating illiteracy, informal education, elementary school, primary high school, senior high school, college or university, and graduate or above), marital status (having a spouse = 1, and no spouse = 0), and individual income (ordinal score from 1 to 23).

#### 2.2.5. Individual Working Factors

Working style represents an individual’s style in approaching work and reflects personality to some degree. Six items were used to measure working style: Likes to try something new or unusual thing/activity, likes to try a unique way to learn something new, likes to use common ways to solve problems, likes to wait for others to start first at work, prepares for future needs, demands or changes in advance, and plans before work.

#### 2.2.6. Organizational Work-Related Factors

The following variables were used to define the working environment:

1. Job satisfaction; scored from 1 to 7, indicating very unsatisfactory to very satisfactory.

2. Underpay; how reasonable you find the salary you are paid by the company/institution, based on five dimensions: Skills, contribution, experience, performance, and responsibility. The rating in five dimensions was scored from 1 to 5, or from very reasonable to very unreasonable. The overall score of the five items (5–25) was used as the score of the degree of feeling underpaid.

3. Interpersonal environment; how you rate the interpersonal relationships in your work setting, that is, relationships between supervisors and staff and relationships among coworkers. Each was scored from 1 to 5, or from very good to very poor.

4. Family-work conflict; how frequently work interferes with family life and how frequently family life interferes with work: Each item was coded from never to always, scored, in total, from 2–10.

5. Discrimination at work; experience of being discriminated at work in the past five years (yes/no).

6. Bullying at work; experience of being bullied at work in the past five years (yes/no).

7. Women’s inequality; agreement that female workers are treated as equal to male workers in six domains: Recruitment, pay, getting higher education degree while working, being an advisor, promotions, and work stability. Each item was scored from 1 (strong agreement) to 5 (strong disagreement). The total score was from 6–30.

#### 2.2.7. Social Work-Related Factors

One variable is the rating of the worsening of wealth disparity in society, scored from 1 (strong disagreement) to 7 (strong agreement). The other variable is the perception that it is difficult finding a good job compared with previous times. The score was from 1 to 7, or strong disagreement to strong agreement.

### 2.3. Analysis

Descriptive analysis, one-way analysis of variance, and linear regression analysis were conducted on the data. The correlation matrix of the variables were listed in the please see Supplementary Material.

## 3. Results

Table 1 shows the results of the descriptive analysis of the sample characteristics. Table 2 shows the age group differences in well-being, exhaustion, stress, and related factors. There were significant differences in self-rated heath across age groups, especially between the group aged 18–39 and older groups, with the younger groups reporting better self-rated health. There were also age differences in psychological health, but the main differences came from the group aged 18–39, who had lower psychological health, and the group aged 55–64, who had better psychological health. Among the three exhaustion variables, there were only significant age differences in physical exhaustion and the group aged 55–64, which were physically exhausted compared with the other groups. Emotional exhaustion and the feeling of barely standing it were nonsignificant across age groups. Younger workers reported higher work stress, being in a physically demanding job, greater self-rated work pressure, not being able to work at home, feeling tired before work, and thinking about work before sleep more than older groups did. The only exception was that the need to work on weekends was more stressful for the group aged 65–74. Younger groups used more new methods, following others less and being more creative at work, than older groups. Younger groups also had worse relationships with coworkers, reported more work-family conflicts, and were more likely to be discriminated against than the older groups but, generally, job satisfaction was not significantly different across age groups. Difficulty finding a good job compared with older cohorts was greater in younger groups than in older groups.

Table 3 shows the association of related factors with three types of exhaustion and total exhaustion according to linear regression models. Age groups were set as an ordinal variable in the models, but age was not significant in models M1 to M4. Factors related to physical exhaustion (M1, R^2^ = 0.304) included being female, greater physical stress, higher self-rated stress, being more tired, being more likely to think about work before sleep, having greater work-family conflict, experiencing greater discrimination against women, and difficult finding a good job. Factors related to emotional exhaustion (M2, R^2^ = 0.308) included being female, lower education, higher self-rated stress, feeling tired, thinking about work more often before sleep, lower job satisfaction, more work-family conflicts, and feeling difficult finding a good job. Factors related to feeling one can no longer stand it (M3, R^2^ = 0.301) included lower individual income, more physical stress, greater self-rated stress, less need to work on weekends, feeling more tired, thinking about work more often before sleep, lower job satisfaction, more work-family conflict, experiencing discrimination at work, and feeling difficult finding a good job. Finally, M4 (R^2^ = 0.397) showed the total exhaustion score predicted by associated factors, with significant factors including being female, lower education, lower individual income, more physical stress, greater self-rated stress, less need to work on weekends, feeling more tired, thinking of work more often before sleep, less job satisfaction, more work-family conflicts, discrimination at work, and feeling difficult finding a good job.

The linear regression models of the association of self-rated and psychological health with related factors in the hierarchies are shown in Table 4 and Table 5, respectively. Table 4 shows the association of self-rated health and associated factors. Age was not significant in M5a but, when other variables were added in M5b to M5e, age (being older) was significantly related to worse self-rated health. Total work stress was not significantly related to self-rated health, but the three types of exhaustion were significantly related to worse self-rated health in M5b to M5e, with emotional exhaustion having a larger coefficient for self-rated health than the other two types of exhaustion. Models M5c to M5e added individual working style, organizational factors, and social factors in the hierarchy to present the ecological effect. Being creative at work and using new methods to solve problems, higher job satisfaction, fewer work-family conflicts, less discrimination against women at work, and feeling difficult finding a good job compared with older cohorts were significantly related to worse self-rated health.

Table 5 shows the hierarchical linear regression of psychological health with demographics, stress and exhaustion, individual working factors, organizational factors, and social factors, from M6a to M6e. Since age shows a reverse-U-shaped relationship with psychological health in Table 2, the age group (ordinal) and its square were both added in the models in Table 5. Age (being older) was related to better psychological health, but age squared was significant when exhaustion and other factors were added from M6b to M5e. This result indicates that being older was related to better psychological health, but being even older offset the protective effect and reduced psychological health; in other words, middle-aged workers had better psychological health than younger and older worker age groups. Total work stress was significantly related to lower psychological health in M6b and M6c, but when organizational factors were added, the effect of work stress was not significant. Three types of exhaustion still had strong effects on worsening psychological health, especially emotional exhaustion, and the inability to stand it anymore in the last model, M6e was closely related to psychological health. Creativity, better job satisfaction, better coworker relationships, and fewer work-family conflicts were related to better psychological health.

## 4. Discussion

This study examined the relations between work stress, exhaustion, and well-being with demographics and working style, organizational, and social factors among workers across age groups. Three types of exhaustion affected self-rated health and psychological health. Individual, organizational, and social factors showed effects on exhaustion and well-being. Being creative at work and new individual working style methods, better job satisfaction, and fewer work-family conflicts were related to both self-rated health and psychological health. Discrimination against women and difficulty finding a good job were related to self-rated health, while coworker relationship quality was related to psychological health. Older age showed a negative linear effect on self-rated health, while age showed a reverse-U-shaped relation with psychological health.

### 4.1. Work Stress, Exhaustion, and Well-Being

Six kinds of work stress were reported and the relations to three types of exhaustions were examined. Physical working stress was related to physical exhaustion and feeling unable to stand it any longer. Self-reports of being stressed, feeling tired, and thinking of work before sleep were also related to all three types of exhaustion. Psychological feelings of work stress being closely related to exhaustion were explained by the stress model [17] and empirical studies. However, stress from unusual shifts or workplaces was not significant, because the respondents were pooled from all types of workers, such that their work characteristics could not be separated. Although work stress was related to exhaustion, it was not significantly related to self-rated health or psychological health. It is possible that the variance is mostly explained by exhaustion or that work stress has only an indirect effect on well-being through exhaustion. Therefore, exhaustion does not necessarily occur under stress if there are fewer risk factors and more protective factors.

### 4.2. Individual Factors in Exhaustion and Well-Being

Creativity was significantly related to both self-rated and psychological health, and using new methods was related to self-rated health when age and other factors were controlled for. Working style is not only about an individual’s personality [21], but is also related to on-the-job training in the organization. Working style can be protective in self-rated health and psychological health, and such training is worth the investment of employers.

Female workers reported higher levels of physical and emotional exhaustion, and lower psychological health than male workers. Female workers might encounter greater work-family conflicts due to gender roles [31], or are more likely to perceive mistreatment in the workplace [56].

### 4.3. Organizational Factors in Exhaustion and Well-Being

Previous research has indicated that organizational factors such as low rewards [15,19], based on the effort–reward model [5], and interpersonal support [6,9,23,24,25] affect exhaustion, and well-being. This study also showed similar findings, but organizational factors had different effects on self-rated health and psychological health. Job satisfaction represents comprehensive organizational influences on self-rated and psychological health. Work-family conflict is related to individual factors too, but it is categorized as an organizational factor in this study. Work-family conflict represents role conflicts and affects well-being [6,27,28,29]. In addition to family support, poorer coworker relationships were significantly related to worse psychological health in this study, whereas underpay was not. The effort–reward imbalance model [5] explains how organizational or family social support and a feeling of belonging, are higher psychological needs and more strongly related to psychological health.

An atmosphere of discrimination against women was also significantly related to poorer self-rated health and greater psychological exhaustion. Even though gender discrimination is forbidden or constrained by law, subtle gender discrimination can still exist [57], and such a women-unfriendly discriminatory atmosphere makes female workers unequal and causes greater exhaustion and lower levels of well-being.

### 4.4. Socail Actors in Exhaustion and Well-Being

Social disparity was not significantly related to exhaustion or well-being. However, greater difficulty finding a good job for current cohorts was related to greater exhaustion and worse self-rated health. This means a cohort ecological transition could exist. Younger cohorts also reported it was more difficult to find a good job than older worker cohorts. One explanation is that the economic recession and social pressure for younger cohorts in the workplace nowadays might not be surmountable by the individual alone, as before. Furthermore, younger workers might need to tolerate worse working conditions than before. The other explanation is that younger cohorts are more vulnerable to work demands than older cohorts and thus feel more easily defeated in work settings. Working hard, having a skill, or obtaining higher education might have been useful strategies for a good life in the past, but younger worker cohorts today may need more help to adapt a rapidly changing world.

### 4.5. Age Differences

Younger workers reported greater work stress and had more work-family conflicts, and more recent discriminatory experiences. It is possible that younger workers are still learning to fit into the working environment or that older workers are more resilient in adapting to a changing environment [58]. Resilience has been suggested as an important factor in reducing burnout at work [7,31,32,48]. In addition, adapting and coping strategies, although not as important as systemic reforms, could help workers in managing their work stress. It is also possible that younger workers face harsher working conditions and older workers have greater autonomy in their work [59].

Older workers had worse self-rated health. However, the age effect was not always linear. In this study, psychological health showed a reversed-U-shape with age; first increasing with age and then declining after middle age. The results indicated that older workers used fewer new methods and less creative ways of dealing with work problems, and had less social support from coworkers than younger workers did. It seems that declining health and creativity with age could affect individuals’ potential to deal with work challenges and offset the psychological resilience of older workers in psychological health. The results also imply that the psychological obstacles for older workers in adaptation to new challenges [60] might not be as great as they imagine. It might be realistic to encourage a vision of active aging for older workers if appropriate organizational interventions are effective. Health promotion and training for workers of all ages to use new methods and creative thinking, transforming older workers’ experience with better working methods, and providing social support from coworker for workers of all ages (including older ones) are potentially beneficial to work outcomes and workers’ well-being in the long run.

### 4.6. Limitations

This study has some limitations. First, the data were secondary data and some of the variables were not available. Second, the differences in occupations could require different working styles and produce different working stresses. Due to the limited number of cases, this study did not intend to compare occupational differences. Third, the data were cross-sectional and the causal relation of the working environment and style with stress, exhaustion, and well-being cannot be confirmed. Only the associations among them could be examined. However, the data contained many work-related variables suitable for exploring associations between work and well-being issues across age groups.

## 5. Conclusions

Individual, organizational, and social factors are related to work exhaustion and well-being under work stress. Older ages showed a negative linear relation with self-rated health, while age showed a reverse-U-shaped relation with psychological health. The resilience of older workers could be an opportunity for the global active aging trend and interventions to support older workers in organizations would be beneficial. Creating a healthy and reasonable working environment through policy is suggested. Future research about useful policy strategies to improve active aging for older workers is suggested.

## Figures and Tables

**Table 1:** Description of the sample (*n* = 1298).

Variables	Mean (SD) or %
**Demographics**	
Age groups (%)	
Age 18–39	48.6%
Age 40–54	31.9%
Age 55–64	15.4%
Age 65–74	3.5%
Age 75+	0.5%
Sex (male %)	56.1%
Education (ordinal 1–7)	5.270 (1.142)
Marital status (having spouse %)	57.2%
Individual Income (ordinal 1–23)	6.060 (3.692)
**Well-being, exhaustion and stress**	
Psychological health	11.000 (2.230)
Self-rated health	2.824 (1.063)
Exhaustion (total)	6.453 (2.541)
Physically exhausted	2.436 (1.032)
Emotionally exhausted	2.270 (0.985)
Can’t stand or hang on anymore	1.745 (0.928)
Work Stress (total)	18.07 (3.92)
Physical demanding	3.008 (1.240)
Having work pressure	3.151 (1.186)
Cannot work at home	4.161 (1.318)
Need to work in weekends	3.119 (1.399)
Feeling tired before work	2.152 (1.073)
Thinking of work while going to sleep	2.459 (1.167)
**Individual working factors**	
Working style: new ways	6.606 (1.780)
Working style: follow others	5.829 (1.494)
Working style: prepare in advance	7.864 (1.235)
Working style: creative	10.731 (2.837)
**Organizational factors**	
Job satisfaction	5.309 (2.666)
Underpay	12.030 (4.420)
Relationship with co-workers	3.730 (1.170)
Work-family conflicts	3.530 (1.630)
Discrimination in work experience (yes %)	13.2%
Bully in work experience (yes %)	7.3%
Women discrimination in work	14.784 (5.233)
**Social factors**	
Disparity in society	6.450 (0.984)
Difficult finding a good job for current cohorts	3.379 (1.178)

**Table 2:** Age group differences in well-being and work factors by one-way ANOVA.

Variables	Age 18–39	Age 40–54	Age 55–64	Age 65–74	Age 75+	Sig.	Post hoc test
	(*n* = 630)	(*n* = 408)	(*n* = 200)	(*n* = 45)	(*n* = 7)		Significant difference
Self-rated health	2.97 (1.07)	2.69 (1.03)	2.79 (1.02)	2.26 (1.10)	2.29 (1.38)	***	(age 18–39) vs. (age 40–54, 65–74)
Psychological health	10.72 (2.21)	11.15 (2.19)	11.63 (2.12)	10.91 (2.53)	10.43 (3.10)	***	(age 18–39) vs. (age55–64)
Exhaustion	6.61 (2.44)	6.52 (2.71)	5.91 (2.40)	6.11 (2.43)	6.43 (4.08)	*	(age 18–39) vs. (age55–64)
Physically exhausted	2.47 (0.99)	2.52 (1.10)	2.18 (098)	2.37 (1.00)	2.43 (1.40)	**	(age 18–39) vs. (age 55–64),
							(age 40–54) vs. (age 55–64)
Emotionally exhausted	2.34 (0.97)	2.23 (1.02)	2.14 (0.94)	2.15 (0.99)	2.43 (1.40)		
Can’t stand it anymore	1.79 (0.92)	1.76 (0.95)	1.60 (0.88)	1.59 (0.86)	1.57 (1.51)		
Work stress (total)	18.79 (3.77)	18.01 (3.95)	16.52 (3.70)	16.29 (3.65)	13.00 (3.37)	***	(age 18–39) vs. (age 40–54, 55–64, 65–74, 75+),
							(age 40–54) vs. (age 55–64)
Stress: Physically demanding	3.04 (1.23)	3.01 (1.25)	2.96 (1.25)	2.96 (1.23)	1.57 (0.79)	*	(age 18–39) vs. (age75+)
Stress: Self-reported stressful in work	3.29 (1.15)	3.24 (1.16)	2.76 (1.17)	2.29 (1.18)	1.71 (1.12)	***	(age 18–39) vs. (age 55–64, 65–74, 75+)
							(age 40–54) vs. (age55–64, 65–74, 75+)
Stress: Cannot work at home	4.38 (1.14)	3.98 (1.44)	3.94 (1.44)	3.76 (1.45)	3.71 (1.50)	***	(age 18–39) vs. (age 40–54, 55–64, 65–74)
Stress: Need to work in weekends	3.12 (1.36)	3.17 (1.38)	2.92 (1.50)	3.61 (1.39)	2.86 (1.77)	*	
Stress: Feeling tired before work	2.41 (1.07)	2.05 (1.04)	1.70 (0.97)	1.70 (087)	1.14 (0.38)	***	(age 18–39) vs. (age 40–54, 55–64, 65–74, 75+),
							(age 40–54) vs. (age 55–64)
Stress: Thinking of work while going to sleep	2.55 (1.16)	2.57 (1.14)	2.25 (1.18)	2.17 (1.29)	2.00 (1.29)	**	(age 18–39) vs. (age 55–64),
							(age 40–54) vs. (age 55–64)
New methods	7.07 (1.61)	6.26 (1.83)	6.13 (1.82)	5.59 (1.66)	4.86 (1.57)	***	(age 18–39) vs. (age 40–54, 55–64, 65–74, 75+),
Follow others	7.07 (1.61)	6.26 (1.83)	6.13 (1.82)	5.59 (1.66)	4.86 1.57)	**	
Prepare in advance	7.79 (1.19)	7.97 (1.21)	7.93 (1.38)	7.70 (1.52)	7.71 (0.76)		
Creative in work	10.76 (2.59)	10.78 (2.98)	10.84 (3.11)	9.59 (3.39)	9.00 (3.00)	*	
Job satisfaction	5.22 (3.69)	5.30 (0.96)	5.54 (0.95)	5.59 (1.05)	5.43 (0.98)		
Underpay	11.99 (4.25)	12.26 (4.51)	11.45 (4.45)	13.05 (5.45)	12.29 (6.18)		
Relationship with co-workers	3.78 (1.17)	3.79 (1.15)	3.53 (1.21)	3.36 (1.08)	3.20 (1.10)	*	
Work-family conflicts	3.62 (1.58)	3.69 (1.76)	3.10 (1.51)	2.69 (1.28)	2.00 (0.00)	***	(age 18–39) vs. (age 55–64, 65–74),
							(age 40–54) vs. (age 55–64, 65–74)
Discrimination	0.15 (0.35)	0.14 (0.35)	0.09 (0.28)	0.04 (0.21)	0.00 (0.00)	*	
Bully	0.08 (0.28)	0.08 (0.27)	0.05 (0.21)	0.04 (0.21)	0.00 (0.00)		
Women inequality	14.72 (5.28)	14.90 (5.33)	14.79 (5.04)	14.50 (4.55)	15.57 (4.54)		
Disparity in society	6.46 (0.96)	6.48 (0.94)	6.41 (1.06)	6.36 (1.22)	5.71 (1.70)		
Difficult finding a good job	3.56 (1.10)	3.31 (1.18)	3.03 (1.26)	3.04 (1.31)	3.17 (0.98)	***	(age 18–39) vs. (age40–54, 55–64)

Note: *n* = 1298. Discrimination and bully experiences were coded as 0/1. Analysis by one-way ANOVA with Scheffe post-hoc test. * *p* <0.05, ** *p* <0.01, *** *p* <0.001.

**Table 3:** Different exhaustion of workers and associated factors by linear regression.

Variable	M1: Physically exhausted B (SE)	M2: Emotionally exhausted B (SE)	M3: Cannot hang on anymore B (SE)	M4: Total Exhaustion B (SE)
Age	0.042 (0.042)	0.030 (0.040)	0.040 (0.037)	0.111 (0.095)
Sex (male)	−0.233 (0.056) ***	−0.188 (0.054) ***	−0.082 (0.050)	−0.503 (0.128) ***
Education	−0.055 (0.035)	−0.071 (0.034) *	−0.058 (0.031)	−0.183 (0.080) *
Marital status (having spouse)	−0.034 (0.059)	−0.062 (0.056)	−0.034 (0.053)	−0.130 (0.134)
Individual income	−0.012 (0.009)	−0.017 (0.009)	−0.019 (0.008) *	−0.047 (0.020) *
Stress: physical	0.172 (0.025) ***	0.033 (0.024)	0.083 (0.022) ***	0.288 (0.056) ***
Stress: stressful	0.143 (0.029) ***	0.149 (0.028) ***	0.107 (0.026) ***	0.400 (0.065) ***
Stress: at home	0.013 (0.023)	0.036 (0.022)	0.031 90.021)	0.080 (0.052)
Stress: weekends	−0.018 (0.021)	−0.027 (0.020)	−0.054 (0.019) **	−0.099 (0.047) *
Stress: tired	0.127 (0.029) ***	0.165 (0.028) ***	0.159 (0.026) ***	0.451 (0.067) ***
Stress: think before sleep	0.081 (0.027) **	0.081 (0.026) **	0.064 (0.024) **	0.226 (0.061) ***
Creative in work	0.017 (0.011)	0.005 (0.011)	0.002 (0.010)	0.023 (0.026)
New methods	0.005 (0.017)	−0.011 (0.016)	−0.011 (0.015)	−0.017 (0.038)
Follow in work	−0.027 (0.018)	0.004 (0.017)	0.021 (0.016)	−0.003 (0.041)
Prepare in work	−0.004 (0.024)	0.012 (0.023)	0.001 (0.022)	0.010 (0.055)
Job satisfaction	−0.020 (0.032)	−0.098 (0.031) **	−0.075 (0.029) *	−0.194 (0.074) **
Underpay	0.007 (0.007)	0.005 (0.007)	0.004 (0.006)	0.017 (0.016)
Co-worker relationship	0.024 (0.025)	0.001 (0.024)	4.631×10^−5^ (0.023)	0.026 (0.058)
Work-family conflicts	0.112 (0.020 ***	0.111 (0.019) ***	0.100 (0.018) ***	0.323 (0.045) ***
Discrimination	0.112 (0.081)	0.131 (0.078)	0.288 (0.073) ***	0.542 (0.185) **
Bully	−0.043 (0.103)	0.068 (0.099)	0.030 (0.093)	0.055 (0.235)
Women discrimination	0.011 (0.005) *	0.002 (0.005)	−0.006 (.005)	0.008 (0.012)
Disparity in society	0.014 (0.027)	−0.003 (0.026)	0.021 (0.025)	0.032 (0.063)
Difficult finding a good job	0.053 (0.023) *	0.062 (0.022) **	0.051 (0.021) *	0.166 (0.053) **
R square	0.304	0.308	0.301	0.397

Note: *n* = 1154. B (SE) stands for beta coefficient (standard error). Categorical variable reference groups: Sex (female), and marital status (no spouse). Constants were omitted. * *p* <0.05, ** *p* <0.01, ** *p* <0.001.

**Table 4:** Self-rated health of workers and association with work stress and exhaustion by linear regression.

Variable	M5a	M5b	M5c	M5d	M5e
	B (SE)	B (SE)	B (SE)	B (SE)	B (SE)
**Individual demographic factors**					
Age	−0.046 (0.049)	−0.103 (0.046) *	−0.102 (0.046) *	−0.104 (0.045) *	−0.111 (0.045) *
Sex (male)	0.163 (0.067) *	0.103 (0.062)	0.088 (0.061)	0.084 (0.061)	0.099 (0.061)
Education	0.080 (0.039) *	0.076 (0.036) *	0.028 (0.037)	0.063 (0.037)	0.076 (0.037) *
Marital status	−0.055 (0.071)	−0.060 (0.065)	−0.061 (0.065)	−0.027 (0.064)	−0.034 (0.064)
Individual income	0.007 (0.010)	0.002 (0.009)	−0.007 (0.009)	−0.009 (0.009)	−0.010 (0.009)
**Exhaustion and stress**					
Total work stress		−0.006 (0.009)	−0.006 (0.009)	0.003 (0.009)	0.002 (0.009)
Physically exhausted		−0.138 (0.039) **	−0.155 (0.039) ***	−0.128 (0.039) **	−0.125 (0.039) **
Emotionally exhausted		−0.200 (0.046) ***	−0.197 (0.045) ***	−0.165 (0.045) ***	−0.159 (0.045) ***
Cannot hang on anymore		−0.152 (0.047) **	−0.140 (0.046) **	−0.126 (0.046) **	−0.121 (0.046) **
**Individual working factors**					
Creative in work			0.044 (0.012) ***	0.038 (0.012) **	0.036 (0.012) **
New methods			0.064 (0.018) **	0.060 (0.018) **	0.059 (0.018) **
Follow in work			−0.012 (0.020)	−0.010 (0.020)	−0.009 (0.020)
Prepare in work			0.044 (0.027)	0.039 (0.026)	0.035 (0.026)
**Organizational factors**					
Job satisfaction				0.081 (0.035) *	0.082 (0.035) *
Underpay				0.000 (0.008)	0.001 (0.008)
Co-worker relationship				−0.037 (0.028)	−0.038 (0.028)
Work-family conflicts				−0.063 (0.022) **	−0.061 (0.022) **
Discrimination experience				−0.042 (0.090)	−0.036 (0.090)
Bully experience				0.139 (0.113)	0.135 (0.113)
Women discrimination				−0.023 (0.006) ***	−0.022 (0.006) ***
**Social factors**					
Disparity in society					−0.004 (0.030)
Difficult finding a good job					−0.070 (0.026) **
**R square**	0.020	0.171	0.207	0.238	0.244

Note: *n* = 1154. B (SE) stands for beta coefficient (standard error). Categorical variable reference groups: Sex (female), and marital status (no spouse). Constants were omitted. * *p* <0.05, ** *p* <0.01, *** *p* <0.001.

**Table 5:** Psychological health of workers and association with work stress and exhaustion by linear regression.

Variable	M6a	M6b	M6c	M6d	M6e
	B (SE)	B (SE)	B (SE)	B (SE)	B (SE)
**Individual demographic factors**					
Age	0.942 (0.377) *	0.955 (0.293) **	0.857 (0.293) **	0.923 (0.289) **	0.905 (0.289) **
Age square	−0.150 (0.084)	−0.202 (0.065) **	−0.189 (0.065) **	−0.203 (0.064) **	−0.200 (0.064) **
Sex (male)	0.164 (0.138)	0.010 (0.109)	0.019 (0.109)	0.041 (0.108)	0.049 (0.108)
Education	−0.047 (0.081)	−0.058 (0.063)	−0.078 (0.065)	0.003 (0.065)	0.009 (0.065)
Marital status	0.180 (0.150)	0.135 (0.117)	0.093 (0.117)	0.126 (0.115)	0.121 (0.116)
**Individual income**	0.038 (0.021)	0.017 (0.016)	0.006 (0.016)	−0.006 (0.017)	−0.006 (0.017)
Exhaustion and stress					
Total work stress		−0.048 (0.016) **	−0.050 (0.016) **	−0.024 90.016)	−0.025 (0.016)
Physically exhausted		−0.208 (0.069) **	−0.228 (0.069) **	−0.194 (0.068) **	−0.193 (0.068) **
Emotionally exhausted		−0.739 (0.080) ***	−0.739 (0.080) ***	−0.665 (0.079) ***	−0.660 (0.079) ***
Cannot hang on anymore		−0.599 (0.081) ***	−0.583 (0.081) ***	−0.529 (0.081) ***	0.528 (0.081) ***
**Individual working factors**					
Creative in work			0.067 (0.022) **	0.056 (0.021) *	0.053 (0.022) *
New methods			−0.054 (0.033)	−0.057 (0.032)	−0.057 (0.032)
Follow in work			−0.051 (0.035)	−0.039 (0.035)	−0.039 (0.035)
Prepare in work			0.047 (0.047)	0.033 (0.046)	0.030 (0.046)
**Organizational factors**					
Job satisfaction				0.215 (0.062) **	0.215 (0.062) **
Underpay				−0.010 (0.013)	−0.010 (0.013)
Co-worker relationship				−0.109 (0.049) *	−0.109 (0.049) *
Work-family conflicts				−0.100 (0.038) **	−0.098 (0.038) *
Discrimination experience				−0.116 (0.158)	−0.110 (0.158)
Bully experience				−0.105 (0.199)	−0.108 (0.200)
Women discrimination				−0.019 (0.010)	−0.019 (0.010)
**Social factors**					
Disparity in society					0.038 (0.053)
Difficult finding a good job					−0.049 (0.045)
**R square**	0.035	0.421	0.430	0.457	0.458

Note: *n* = 1154. B (SE) stands for beta coefficient (standard error). Categorical variable reference groups: Sex (female), and marital status (no spouse). Constants were omitted. * *p* <0.05, ** *p* <0.01, *** *p* <0.001.

## Supplementary Materials

The following are available online at http://www.mdpi.com/1660-4601/16/1/50/s1, Table S1: Correlation matrix of the variables.
