# Supplementary material for: Age Differences in Work Stress, Exhaustion, Well-Being, and Related Factors From an Ecological Perspective
Source: Int J Environ Res Public Health. 2018 Dec 25;16(1):50. doi: 10.3390/ijerph16010050 (PMC6338997; doi:10.3390/ijerph16010050)
Supplement: Supplementary file 1 [file ijerph-16-00050-s001.pdf]

Supplementary Table 1. Correlation matrix of the variables.

Title	Sex	Age	Education	Marital status	Income	Child	Psychological health	Exhaustion	Self-rated health	Happy	Total work stress	Work stress: physical	Work stress: stressful	Work stress: at home	Work stress: weekends	Work stress: tired	Work stress: think before sleep	New methods	Follow	Preparation	Creative	Job satisfaction	Underpay	Coworker relationship	Work-family conflict	Discrimination	Bully	Women discrimination	Disparity in society	Difficult finding a job
Sex	1																													
Age	0.072 **	1																												
Edu	−0.004	−0.544 **	1																											
Marital status	−0.002	0.488 **	−0.230 **	1																										
Income	0.218 **	0.087 **	0.312 **	0.144 **	1																									
Child	−0.027	0.022	−0.056 *	0.320 **	0.033	1																								
Psychological health	0.080 **	0.132 **	−0.031	0.095 **	0.103 **	−0.014	1																							
Exhaustion	−0.118 **	−0.085 **	0.005	−0.047	−0.074 **	0.036	−0.620 **	1																						
Self-rated health	0.109 **	−0.139 **	0.167 **	−0.061 *	0.079 **	−0.011	0.420 **	−0.397 **	1																					
Happy	0.017	−0.026	0.036	−0.013	0.044	−0.017	0.112 **	−0.103 **	0.067 *	1																				
Total work stress	0.071 *	−0.224 **	0.070 *	−0.106 **	0.000	0.005	−0.372 **	0.481 **	−0.181 **	−0.091 **	1																			
Work stress: physical	0.127 **	−0.051	−0.223 **	−0.036	−0.176 **	0.027	−0.124 **	0.254 **	−0.049	−0.077 **	0.596 **	1																		
Work stress: stressful	0.028	−0.152 **	0.284 **	−0.020	0.251 **	0.029	−0.300 **	0.387 **	−0.130 **	−0.012	0.612 **	0.151 **	1																	
Work stress: at home	−0.019	−0.175 **	0.027	−0.136 **	−0.119 **	−0.011	−0.065 *	0.057 *	−0.023	−0.085 **	0.294 **	0.100 **	−0.039	1																
Work stress: weekends	0.071 *	−0.012	−0.122 **	−0.024	−0.060 *	−0.014	−0.064 *	0.117 **	−0.073 **	−0.003	0.513 **	0.299 **	0.130 **	−0.174 **	1															
Work stress: tired	−0.020	−0.264 **	0.173 **	−0.141 **	−0.023	−0.045	−0.415 **	0.445 **	−0.180 **	−0.111 **	0.621 **	0.199 **	0.355 **	0.095 **	0.151 **	1														
Work stress: thinking before sleep	0.026	−0.071 *	0.132 **	0.031	0.168 **	0.043	−0.292 **	0.346 **	−0.152 **	−0.018	0.588 **	0.142 **	0.447 **	−0.083 **	0.145 **	0.312 **	1													
New methods	0.050	−0.278 **	0.287 **	−0.167 **	0.099 **	−0.076 **	−0.016	0.014	0.177 **	0.055	0.039	−0.044	0.106 **	−0.063 *	−0.007	0.070 *	0.084 **	1												
Follow	0.059 *	−0.106 **	0.099 **	−0.035	−0.021	−0.056 *	−0.072 *	0.054	−0.009	−0.009	0.065 *	0.014	0.025	0.059 *	0.004	0.152 **	−0.029	0.069 *	1											
Preparation	0.022	0.029	0.162 **	0.084 **	0.181 **	−0.002	0.050	0.020	0.110 **	0.017	0.030	−0.055 *	0.124 **	−0.124 **	0.037	−0.025	0.154 **	0.212 **	0.013	1										
Creative	0.068 *	−0.015	0.218 **	0.065 *	0.266 **	−0.010	0.081 **	0.010	0.168 **	0.070 *	0.046	−0.058 *	0.213 **	−0.174 **	0.024	−0.060 *	0.217 **	0.318 **	−0.057 *	0.341 **	1									
Job satisfaction	−0.035	0.056 *	−0.041	0.067 *	0.058 *	0.040	0.084 **	−0.036	0.056 *	0.050	−0.092 **	−0.069 *	−0.077 **	−0.040	−0.001	−0.084 **	−0.037	0.003	−0.024	0.015	0.056 *	1								
Underpay	−0.027	−0.003	−0.012	−0.043	−0.202 **	−0.013	−0.229 **	0.222 **	−0.120 **	−0.009	0.257 **	0.133 **	0.162 **	0.032	0.131 **	0.236 **	0.150 **	−0.043	−0.004	−0.020	−0.037	−0.398 **	1							
Coworker relationship	0.016	−0.070 *	0.077 **	−0.040	−0.079 **	0.005	−0.259 **	0.198 **	−0.170 **	−0.059 *	0.186 **	0.071 *	0.150 **	0.104 **	0.008	0.222 **	0.070 *	−0.057	0.127 **	−0.114 **	−0.127 **	−0.101 **	0.240 **	1						

Work-family conflict	−0.005	−0.094 **	0.220 **	0.060 *	0.190 **	0.114 **	−0.331 **	0.413 **	−0.166 **	−0.016	0.397 **	0.101 **	0.445 **	−0.092 **	0.175 **	0.343 **	0.354 **	0.086 **	0.083 **	0.138 **	0.183 **	0.003	0.194 **	0.193 **	1					
Discrimination	−0.050	−0.060 *	0.034	−0.059 *	−0.048	0.041	−0.186 **	0.221 **	−0.107 **	0.021	0.175 **	0.075 **	0.160 **	0.056 *	0.034	0.163 **	0.090 **	0.035	0.008	−0.002	−0.009	0.018	0.140 **	0.188 **	0.170 **	1				
Bully	−0.038	−0.065 *	0.045	−0.062 *	−0.020	−0.001	−0.132 **	0.142 **	−0.037	0.067 *	0.139 **	0.051	0.142 **	0.067 *	0.031	0.087 **	0.086 **	0.056 *	0.046	0.024	0.033	−0.048	0.071 *	0.135 **	0.113 **	0.293 **	1			
Women discrimination	−0.106 **	0.028	0.012	0.030	0.018	−0.007	−0.077 **	0.047	−0.157 **	0.017	−0.008	−0.059 *	0.013	0.001	−0.001	0.027	0.008	−0.081 **	−0.067 *	−0.051	−0.090 **	−0.027	0.052	0.040	0.021	0.048	−0.005	1		
Disparity	0.024	−0.007	0.070 *	0.020	0.009	0.024	−0.032	0.075 **	−0.006	0.007	0.089 **	0.059 *	0.110 **	−0.030	0.058 *	0.065 *	0.037	0.019	0.047	0.047	0.046	−0.001	0.098 **	0.000	0.058 *	0.030	0.020	0.071 *	1	
Difficult finding a job	0.050	−0.162 **	0.181 **	−0.094**	0.002	−0.037	−0.127 **	0.136 **	−0.083 **	0.007	0.093 **	−0.011	0.085 **	−0.030	0.039	0.144 **	0.088 **	0.017	0.059 *	−0.063 *	−0.048	0.000	0.069 *	0.062 *	0.123 **	0.072 **	0.018	0.056	0.166 **	1

2 Note: Pearson’s correlation matrix. * $p < 0.05$, ** $p < 0.01$. *** $p < 0.001$.
